# Exercise load monitoring: integrated approaches to advance the individualisation of exercise oncology

**DOI:** 10.1136/bmjsem-2021-001134

**Published:** 2021-08-30

**Authors:** Stephen J Carter, Marissa N Baranauskas, Tarah J Ballinger, Laura Q Rogers, Kathy D Miller, Dustin C Nabhan

**Affiliations:** 1 Department of Kinesiology, Indiana University Bloomington, Bloomington, Indiana, USA; 2 Indiana University Melvin and Bren Simon Comprehensive Cancer Center, Indianapolis, Indiana, USA; 3 Department of Medicine, Indiana University School of Medicine, Indianapolis, Indiana, USA; 4 Department of Medicine, University of Alabama at Birmingham, Birmingham, Alabama, USA; 5 United States Olympic & Paralympic Committee, Colorado Springs, Colorado, USA; 6 Norwegian School of Sport Sciences, Oslo, Norway

**Keywords:** aging, fatigue, obesity, performance, physical activity

## Abstract

Whether slowing disease progression or combatting the ills of advancing age, the extensive utility of exercise training has contributed to the outright declaration by the American College of Sports Medicine that ‘exercise is medicine’. Consistent with general framework of adaptation, the advantages of exercise training are indiscriminate—benefitting even the most susceptible clinical populations. Still, the benefit of exercise training presupposes healthy adaptation wherein progressive overload matches sufficient recovery. Indeed, a difference exists between healthy adaptation and non-functional over-reaching (ie, when internal/external load exceeds recovery capacity)—a difference that may be blurred by cancer treatment and/or comorbidity. Recent advances in smartwatches make them ideally suited to non-invasively monitor the physiological stresses to exercise training. Resolving whether individuals are successfully adapting to exercise training via load monitoring bears clinical and practical relevance. While behaviour-change research aims to identify positive constructs of exercise adherence, further attention is needed to uncover how to optimise exercise prescription among cancer populations. Herein, we briefly discuss the constituents of exercise load monitoring, present examples of internal and external load and consider how such practices can be applied to cancer populations.

**Key messages**

**What is already known**

- The systemic benefits of exercise training are irrefutable.
- A difference exists between healthy adaptation and non-functional over-reaching (ie, when internal/external load exceeds recovery capacity)—a difference that may be blurred by cancer treatment and/or comorbidity.

**What are the new findings**

- Smartwatches are ideally suited to non-invasively monitor the physiological stresses to exercise training.
- Resolving whether individuals are successfully adapting to exercise training via load monitoring bears clinical and practical relevance.

## Introduction

The benefit of exercise training presupposes healthy adaptation wherein progressive overload matches sufficient recovery. However, one of the most challenging barriers of exercise prescription is knowing how and when to modulate frequency, intensity and duration for continued improvement (ie, effective exercise dose).^1^ Although numerous entities advocate general exercise recommendations, what is less clear is how to identify individual adaptation to a given training progression. This becomes increasingly critical among cancer populations where healthy adaptation may be weakened by the direct and/or indirect effects of both the disease and its treatment.

In the last 20 years, considerable momentum has been achieved within in the field of exercise oncology—now recognised as subdiscipline of oncology research.^2^ It was not long ago when exercise avoidance, particularly among patients undergoing cancer treatment, was commonplace. Not until the seminal work of MacVicar *et al* were some of the concerns surrounding exercise training in cancer patients assuaged.^3^ While numerous randomised clinical trials are actively evaluating the utility of exercise training, understanding exercise response variability remains a challenge. Even the term ‘effective exercise dose’ may differ depending on the outcome of interest. For instance, resistance exercise may be less effective for improving anxiety or depression though beneficial for lymphedema.^4^ Given the complexities involving cancer type and treatment, cancer populations are uniquely confronted by a myriad of side effects including pain, fatigue, cognitive alterations, sleep disturbance and systemic deconditioning. The prevalence of such comorbidities can interfere with optimal exercise recovery, which in turn underscores the necessity for advancing the integration of exercise load monitoring (see online supplemental table 1).

10.1136/bmjsem-2021-001134.supp1 Supplementary data

From a practical perspective, recent advances in smartwatches make them ideally suited to non-invasively monitor the physiological stresses to exercise training. Such an approach extends the reach beyond laboratory settings and the need for expensive instruments to evaluate progress. Given the current challenges attributed to the COVID-19 pandemic, having remote access to individual exercise training responses is an attractive option to strengthen home-based exercise. Resolving the nuances of healthy adaptation and recovery will assuredly influence exercise prescription with related effects on injury risk and exercise adherence as well. The exercise training dose–response relationship is akin to a pharmacological investigation evaluating the efficacy of a medication. Indeed, a difference exists between healthy adaptation and non-functional over-reaching (ie, when internal/external load exceeds recovery capacity)^5^—a difference that may be blurred by cancer treatment and/or comorbidity.

Though the absolute workload is appreciably higher among athletic populations, the same principles of training, recovery and adaptation apply to cancer populations. While the implementation of exercise load monitoring may vary, the importance of individualising such an approach cannot be overstated. Since age, cardiorespiratory fitness and non-exercise stressors all contribute to the rate of recovery, it is unsurprising to observe large within-person/between-person differences emerge regarding exercise tolerance and subsequent adaptations. Therefore, to promote further sophistication in the field of exercise oncology, the present viewpoint briefly discusses the constituents of exercise load monitoring, presents examples of internal and external load and considers how such practices can be applied to cancer populations.

### Defining exercise load

It is customary to delineate the quantification of exercise training load into internal and external constructs. Internal training load reflects physiological (eg, heart rate (HR)) and psychological (eg, rating-of-perceived exertion (RPE)) stressors, whereas external training load represents metrics of work performed such as distance travelled, steps per day or repetition counts. Consensus indicates a combined approach yields a clearer perspective about the inherent oscillations involving exercise adaptation and recovery.^6^ For instance, distance travelled (external load) may not fluctuate during repeated exposure to a 6-minute walk test, however, measures of HR and/or RPE (internal) may reveal a mismatch between effort and performance—thus providing information about the status of adaption for an individual. Certainly, the dissociation between internal and external loads may expose whether the exercise dose is appropriate for the individual’s needs.

### Internal load monitoring

#### Heart rate

Wearable health monitoring technologies including smartwatches have become a familiar sight. Owing to the non-invasive capabilities of photoplethysmography, infrared light is used to measure the volumetric variations of blood circulation—making it possible to measure and record HR in real time. At the onset of exercise, sympathetic activity rises to produce an intensity-dependent increase in HR. As such, HR and oxygen uptake (V̇O_2_) share a linear relationship at submaximal intensities. Given the combined effects of blood volume expansion, increased stroke volume and heightened parasympathetic outflow, one of the most prominent phenotypic attributes of exercise training is a lowered HR at rest and during exercise. Since HR fluctuates daily, a single measurement provides little useful interpretive information such that repeated measurements and consideration for adequate hydration are essential. Unlike the transient changes in HR over the course of a day, resting HR when measured under standardised conditions is thought to reflect global cardiovascular health. Thus, changes in resting HR over days may be indicative of physiologic or psychologic stressors, whereas alterations over weeks may represent increased (or decreased) cardiorespiratory fitness.^7^

#### Rating of perceived exertion

One of the more traditional approaches to measuring internal load is RPE. The conceptual basis of the RPE Scale conveys sensory feedback about the strenuousness of a physical task, yet the measure is also known to be influenced by emotion and pain. While RPE positively correlates with exercise intensity, there are instances when comorbidity can overshadow sensations arising from muscular work—thus affecting rating behaviour.^8^ From a monitoring perspective, the RPE Scale is a convenient tool that can be implemented during or after exercise training—the latter referred to as session RPE.^9^ Given the prevalence of fatigue and fatigability among cancer populations, coupling HR with an RPE measure (HR:RPE ratio) during a submaximal walking task may reveal short-term/long-term fluctuations about exercise adaptation. For instance, the internal load of a cancer survivor exhibiting a blunted HR response with an increased RPE may be experiencing something entirely different compared with an ‘expected’ HR response and corresponding RPE.

### External load monitoring

Numerous technologies exist to quantify the external work performed by an individual. Extensive use of accelerometry and global positioning equipped smartwatches present feasible opportunities to better understand performance indicators over days, weeks and months. A significant advantage to wearable technologies is the continuous assessment of distance travelled or steps taken during exercise training as well as during routine daily living. Certainly, maladaptation to exercise is not solely a function of physical exertion per se and instead is also influenced by everyday living including diet^10^ and sleep patterns.^11^ Along the same lines of standardising the measurement of HR, the approach used to monitor external work should be consistent in a way that illuminates progress or stagnation. Determination of distance travelled during a 6-minute walk test offers clinical utility concerning functional status and walking autonomy.^12^ Mobile app-based measurement of HR during a 6-minute walk test could yield valid measures reflective of exercise adaptation while circumventing laboratory-based examination.^13^

### Internal: external load ratio

Assimilating the internal and external load ratio can be used to evaluate the psychophysiological state of an individual in response to exercise training. Generally, positive adaption may be surmised should the individual exhibit a reduced HR and/or RPE compared with pre-exercise training levels in response to a standard measure of external load. Recurrent assessments over a training progression should be used to inform changes in exercise training volume. Regrettably though, a prevailing methodological limitation to exercise training studies among cancer populations involves the scarcity of information about exercise prescription (ie, frequency, intensity, duration) modification. This common shortcoming is thought to interfere with study reproducibility, interpretation and cross-study integration^14^—thus underscoring the essential role for not only exercise load monitoring but also for refinement in how exercise dose/tolerance is reported.

### Analysing exercise load data

Access to wearables can expedite the collection of internal and external load data, however, having a system in place to make meaningful inferences about adaptive responses can be challenging. Numerous research publications have endeavoured to extract the most from exercise training in elite athletes—yet such efforts are still gaining traction in cancer populations.^15–17^ Functionality of the miniaturised biosensors housed within smartwatches permits wireless, non-invasive and autonomous communication with smartphones. The growing number of consumer-based health analytic platforms (eg, Habit Dash) provides a convenient way to integrate exercise and physical activity data. Such tools have the advantage of assembling a comprehensive appraisal of how an individual may be responding to exercise. Likewise, advances in ecological momentary assessment tools enable research scientists to capture real-time perceptual data.

### Conclusions

Resolving whether individuals are successfully adapting to exercise training via load monitoring bears clinical and practical relevance. While behaviour-change research aims to identify positive constructs of exercise adherence, further attention is needed to uncover how to optimise exercise prescription among cancer populations. Consideration for measurement error notwithstanding, there is need to identify early signs of maladaptation, including the smallest worthwhile change,^18^ wherein practitioners can modify the exercise prescription to promote healthy adaptation.
