# Supplementary material for: Exercise load monitoring: integrated approaches to advance the individualisation of exercise oncology
Source: BMJ Open Sport Exerc Med. 2021 Aug 30;7(3):e001134. doi: 10.1136/bmjsem-2021-001134 (PMC8407211; doi:10.1136/bmjsem-2021-001134)
Supplement: Supplementary data [file bmjsem-2021-001134supp001.pdf]

Table 1. List of key terms.

Terms	Working Definition
Adaptation	Behavioral, physiological, and structural changes in response to exercise training.
Cardiorespiratory fitness[1]	General ability to perform large muscle, dynamic moderate-to-vigorous exercise for prolonged periods.
Exercise training[1]	Physical activity consisting of planned, structured, and repetitive bodily movement performed to improve and/or maintain one or more components of physical fitness.
Exercise prescription[1]	Progression of exercise training modulated through changes in frequency, intensity, time, type, and volume.
Exercise tolerance	Reflects the voluntary ability to endure sustained exercise.
External load	In the context of exercise, reflects the cumulative workload performed. Example: steps per day or distance traveled.
Internal load	In the context of exercise, reflects the cumulative physiological and psychological burden. Example: rating-of-perceived exertion or heart rate.
Non-exercise stressors	Includes such variables as diet and sleep that may influence adaptation.
Non-functional overreaching	When internal and/or external load of exercise training exceeds recovery capacity. Often linked with stagnation or decreased performance.
Overload	Progressively increasing the demands of exercise training to trigger adaptation.
Principles of exercise training	Multidimensional, includes specificity, overload, individualization, and reversibility.
(Exercise) Recovery	Describes the global readiness in either the short- and/ or long-term to exercise train.
Smart watches	Wearable device with multiple applications that autonomously collect internal and external load data.

[1]In accordance with the American College of Sports Medicine.
